# When a diet is followed too strictly. Scurvy – An old disease in a modern gut: A case report

**DOI:** 10.1097/MD.0000000000043688

**Published:** 2025-08-01

**Authors:** Léa Guarino, Olivier Chatelanat, Pablo Gressot, Christophe Larpin, Jacques Serratrice, Matteo Coen

**Affiliations:** a Division of General Internal Medicine, Department of Medicine, Geneva University Hospitals, Geneva, Switzerland; b Department of Gastroenterology and Hepatology, University Hospitals of Geneva Faculty of Medicine, Geneva, Switzerland; c Unit of Development and Research in Medical Education (UDREM), Faculty of Medicine, University of Geneva, Geneva, Switzerland.

**Keywords:** bruising, case report, Crohn disease, ecchymosis, vitamin C deficiency

## Abstract

**Rationale:**

This case highlights the importance of considering a wide range of possible diagnoses when faced with unexplained hemorrhagic symptoms. When standard investigations fail to identify a clear cause, it is essential to conduct a detailed dietary history. This can lead to the diagnosis of scurvy, a reversible vitamin C deficiency that is often overlooked in populations at risk.

**Patient concerns:**

The patient is a 55-year-old man with well-controlled Crohn disease who presented with unexplained bilateral leg pain and extensive hematomas on his lower limbs, significantly affecting his mobility and raising concerns about a serious condition.

**Diagnoses:**

The final diagnosis was scurvy, resulting from a chronic deficiency of vitamin C due to a severely limited diet that lacked fruits and vegetables. Initially, differential diagnoses included deep vein thrombosis and drug-induced coagulopathy. However, a physical examination revealed follicular purpura, and the identified dietary restrictions ultimately led us to the correct diagnosis and enabled effective treatment.

**Interventions:**

Vitamin C supplementation resulted in a rapid improvement in the patient’s condition.

**Outcomes:**

The patient experienced significant recovery following vitamin C supplementation.

**Lessons:**

This case highlights the importance of recognizing nutritional deficiencies in modern medicine, especially for patients with chronic illnesses. It demonstrates that classic diseases, such as scurvy, can still occur and be overlooked if comprehensive patient histories and thorough examinations are not conducted. Timely identification and vitamin C supplementation resulted in a rapid improvement in the patient’s condition, underscoring the importance of considering nutritional factors in unexplained bleeding disorders.

## 1. Introduction

Scurvy is a disease traditionally associated with maritime voyages and malnutrition, often regarded as a condition of the past. However, it still plays a rare yet important role as a differential diagnosis in modern clinical settings, especially among individuals with restrictive diets or malabsorptive disorders. This case report highlights an unusual presentation of scurvy in a patient with Crohn disease who experienced spontaneous hematomas in his lower limbs. Detailed investigations ultimately revealed a severe vitamin C deficiency caused by his diet. This case emphasizes the necessity of considering nutritional deficiencies like scurvy in patients who present with unexplained bleeding, even in developed countries.

## 2. Case presentation

**Figure 1.**
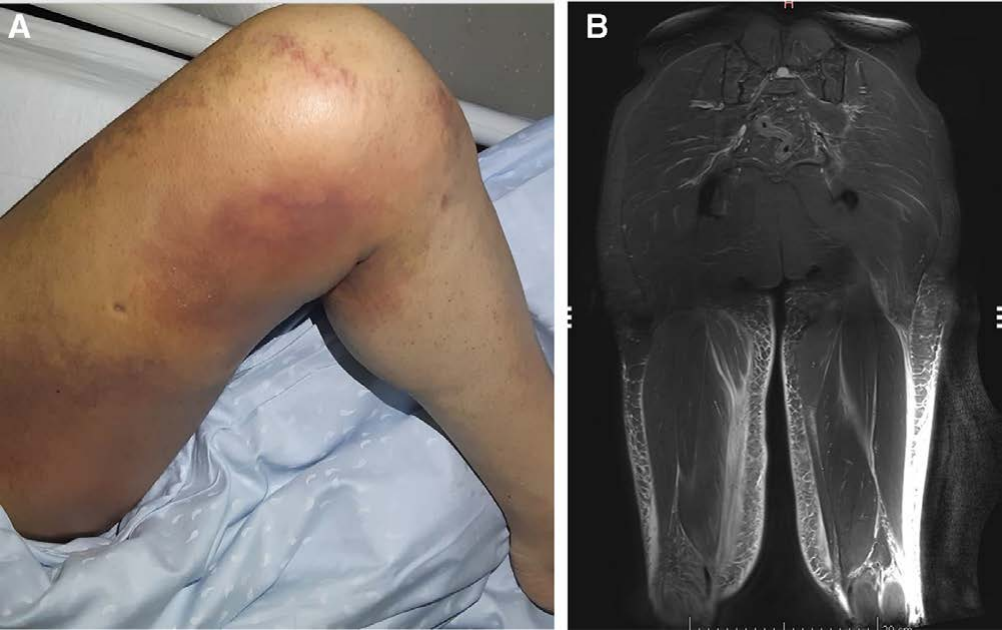
(A) Picture of the lower limbs showing both edema and hematomas. (B) T1-weighted MRI without contrast showing a bilateral extensive fluid infiltration of the skin and subcutaneous tissues, extending to muscular aponeuroses. MRI = magnetic resonance imaging.

A 55-year-old man with well-controlled Crohn disease, treated with azathioprine, was referred to our department due to extensive hematomas affecting both lower limbs (Fig. 1A). In the days leading up to his admission, he visited the emergency department multiple times, complaining of persistent leg pain. He denied experiencing any recent trauma or unusual physical exertion. To rule out deep vein thrombosis, a lower limb ultrasound and a chest CT scan were performed; both tests yielded unremarkable results. Upon admission, azathioprine was discontinued due to concerns about a potential drug-induced cause. Physical examination revealed mild bilateral lower limb edema and extensive ecchymosis, predominantly on the inner and posterior aspects of the thighs, extending down to the feet. Muscle strength was preserved and appeared normal in both lower limbs, with no signs of systemic bleeding or other hemorrhagic manifestations.

Initial laboratory investigations revealed hyperregenerative anemia (hemoglobin level of 6.7 g/dL and an elevated reticulocyte count of 17.86 G/L), necessitating a red blood cell transfusion. The platelet count was within the normal range at 239 G/L. Coagulation studies were unremarkable (prothrombin time >100%, normal <70%; partial thromboplastin time: 34.1 seconds, normal range: 26–37 seconds). No active bleeding was detected during an upper gastrointestinal endoscopy or abdominal computed tomography. C-reactive protein was elevated (85 mg/L; reference <5 mg/L), and creatine phosphokinase remained within normal limits.

Magnetic resonance imaging of the lower limbs was initially interpreted as showing muscle tears with associated hemorrhage. However, a subsequent reevaluation clarified that there was hemorrhagic infiltration of the aponeuroses, without evidence of muscle tears or structural lesions (Fig. 1B).

Given this finding, an extended coagulation workup (including assessments of clotting factors, von Willebrand factor and its activity, and platelet function) was performed, all of which returned normal results.

**Figure 2.**
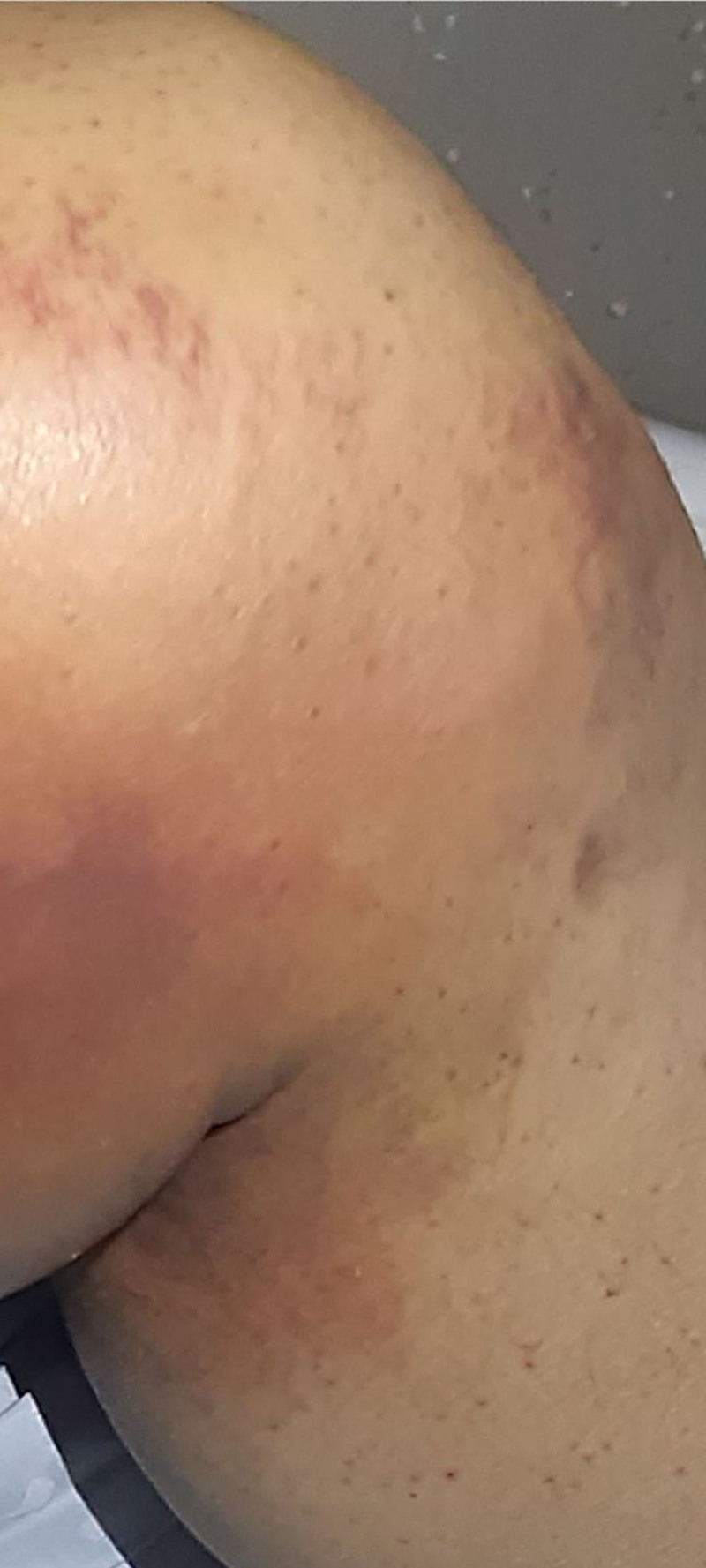
Detail of the left leg showing follicular purpura.

A new and careful examination of the lower limbs revealed follicular purpura associated with hematomas (Fig. 2), prompting consideration of scurvy as a potential underlying cause. During a thorough history-taking, the patient reported a very limited diet consisting of only 5 foods, with no fruit or vegetable intake for over a year, to control the symptoms of his Crohn disease.

On the fifth day of admission, the vitamin C levels were measured and found to be at the lower limit of normal (18.3 µmol/L; reference range: 17.0 < *N* < 85). A presumptive diagnosis of scurvy was established, and a treatment plan of vitamin C supplementation was initiated (ascorbic acid 1000 mg/day for 5 days, followed by 500 mg twice daily for 1 month), alongside dietary modifications. This approach led to a rapid improvement in hemoglobin levels and a progressive resolution of the hematomas.

Azathioprine was successfully reintroduced after a 1-month hiatus with no adverse events. At follow-up, the patient remained asymptomatic and in good health, expressing an intention to maintain these dietary changes to support long-term health and prevent the recurrence of symptoms.

## 3. Discussion

In our patient, the presence of underlying Crohn disease, treated with azathioprine, raises the possibility of side effects.^[1]^ Several cases of acquired von Willebrand disease have been reported in individuals with Crohn disease; however, the normal results from the tests conducted on our patient allowed us to rule out this hypothesis.^[2,3]^ Additionally, azathioprine can reduce factor V activity^[4]^ and may cause thrombocytopenia due to bone marrow suppression.^[2]^ Nevertheless, both the platelet count and coagulation studies were normal. Upon revisiting the patient’s history, particularly the dietary assessment, and carefully examining the lower limbs, we were directed toward a diagnosis of scurvy.

Although the patient’s vitamin C levels were at the lower limit of normal, it is essential to note that both the collagen-related abnormalities linked to scurvy and circulating vitamin C levels can improve rapidly after supplementation begins.^[5,6]^ This likely applied to our patient, whose vitamin C levels were measured 5 days after reintroducing a balanced diet.

## 4. Conclusion

This case highlights the necessity of including scurvy in the differential diagnosis of unexplained hemorrhagic manifestations, even in contemporary clinical practice. Although often viewed as a historical condition, vitamin C deficiency is still a relevant and reversible etiology in suitable clinical contexts.

## Author contributions

**Conceptualization:** Léa Guarino, Olivier Chatelanat, Pablo Gressot, Christophe Larpin, Jacques Serratrice.

**Data curation:** Léa Guarino, Jacques Serratrice.

**Supervision:** Jacques Serratrice, Matteo Coen.

**Validation:** Jacques Serratrice, Matteo Coen.

**Writing – original draft:** Léa Guarino.

**Writing – review & editing:** Olivier Chatelanat, Pablo Gressot, Christophe Larpin, Jacques Serratrice, Matteo Coen.
